# *In situ *gastrointestinal protection against anthrax edema toxin by single-chain antibody fragment producing lactobacilli

**DOI:** 10.1186/1472-6750-11-126

**Published:** 2011-12-20

**Authors:** Kasper Krogh Andersen, Harold Marcotte, Beatriz Álvarez, Prosper N Boyaka, Lennart Hammarström

**Affiliations:** 1 Division of Clinical Immunology and Transfusion Medicine, Department of Laboratory Medicine, Karolinska Institutet at Karolinska University Hospital Huddinge, SE-141 86 Stockholm, Sweden; 2 Department of Veterinary Biosciences, VMAB Room 345, 1900 Coffey Road, Ohio State University, Columbus, OH 43210, USA

## Abstract

**Background**

Anthrax is caused by the bacterium *Bacillus anthracis *and is regarded as one of the most prominent bioterrorism threats. Anthrax toxicity is induced by the tripartite toxin complex, composed of the receptor-binding anthrax protective antigen and the two enzymatic subunits, lethal factor and edema factor. Recombinant lactobacilli have previously been used to deliver antibody fragments directed against surface epitopes of a variety of pathogens, including *Streptococcus mutans, Porphyromonas gingivalis*, and rotavirus. Here, we addressed whether or not anthrax toxins could be targeted and neutralised in the gastrointestinal tract by lactobacilli producing recombinant antibody fragments as a model system for toxin neutralisation in the gastrointestinal lumen.

**Results**

The neutralising anti-PA scFv, 1H, was expressed in *L. paracasei *as a secreted protein, a cell wall-anchored protein or both secreted and wall-anchored protein. Cell wall display on lactobacilli and PA binding of the anchored constructs was confirmed by flow cytometry analysis. Binding of secreted or attached scFv produced by lactobacilli to PA were verified by ELISA. Both construct were able to protect macrophages in an *in vitro *cytotoxicity assay. Finally, lactobacilli producing the cell wall attached scFv were able to neutralise the activity of anthrax edema toxin in the GI tract of mice, *in vivo*.

**Conclusion**

We have developed lactobacilli expressing a neutralising scFv fragment against the PA antigen of the anthrax toxin, which can provide protection against anthrax toxins both *in vitro *and *in vivo*. Utilising engineered lactobacilli therapeutically for neutralising toxins in the gastrointestinal tract can potential be expanded to provide protection against a range of additional gastrointestinal pathogens. The ability of lactobacilli to colonise the gastrointestinal tract may allow the system to be used both prophylactically and therapeutically.

## Background

Spores of *Bacillus anthracis *have for long been regarded as one of the most powerful bioterrorism threats due to their stability and high lethality [1]. The spores can be easily produced and stockpiled in large quantities, using simple microbial techniques by people having access to a virulent strain and incentive to be exposed to the risk connected with its propagation and handling. Previous deliberate spread of anthrax spores as agent of biowarfare has been as aerosol. However, they could also be disseminated through the food or water supply for targeting of the gastrointestinal tract.

Anthrax infections fall into three different categories, reflecting the route of entry; inhalational, gastrointestinal or cutaneous in order of severity of the infection. With regard to bioterrorism, the most realistic mode of mass exposure includes inhalational or gastrointestinal infections. Conceptually, the idea of targeting the food supply is not new [2] and a few records of planned use of anthrax spores for deliberately targeting the oral route exist [3,4]. However, relatively little is known about the pathophysiology of gastrointestinal anthrax, despite its prevalence in ruminant livestock. Initial infection is established in the Peyer''s Patches throughout the small intestine, eventually leading to systemic infection by spreading to the draining jejunal lymph nodes, the spleen and, finally, the lungs. Gastrointestinal infection by *B. anthracis *preferentially occurs after abrasions in the mucosa but can also occur in the absence of damage in which case infection propagation is slower [5]. Natural occurrence of human gastrointestinal anthrax in the western world is rare due to the high standard of the food supply chain but is more common than inhalational anthrax in the developing world [6].

The pathogenesis of *B. anthracis *is due to the product of three plasmid encoded (pXO1) toxicity genes; *pagA *(PA), *lef *(LF) and *cya *(EF) expressing a tripartite protein complex, causing the lethal symptoms associated with anthrax. The protective antigen (PA) combines with the lethal factor (LF) and edema factor (EF) to form the lethal toxin (LT) and edema toxin (ET) respectively [7]. PA is the component affording binding to either of two receptors, the tumor endothelial marker 8 (TEM8) and the capillary morphogenesis 2 (CMG2) [8]. The receptor bound PA is proteolytically activated facilitating oligomerization of PA into a heptameric prepore structure, forming the binding sites for LF and EF. The complete toxin complex is endocytosed and, upon acidification of the early endosome, the prepore undergoes conformational change whereby LF and EF are translocated into the cytosol (for review see [9]). LF is a metalloprotease cleaving MAPK (mitogen-activated protein kinase) kinases [10], inactivating MAPK signaling pathways and inducing an atypical vascular collapse in mice [11]. EF is a calmodulin-dependent adenylate cyclase which increases cyclic AMP levels in cells and induces extensive intestinal fluid accumulation and hemorrhaging lesions [12,13]. Both active and passive vaccination strategies against anthrax have previously been attempted and directed primarily towards inactivation of the toxin components, where PA is the dominant immunogen, and several neutralising antibodies binding to epitopes blocking the binding to its receptors have previously been developed [14,15].

Anthrax Vaccine Adsorbed (AVA) is at present the only vaccine licensed for use in the United States for prophylactic treatment against anthrax. However, the vaccine requires multiple injections over 12-18 months in order to be effective [16] and due to its cost and side effects, therapeutic treatment is currently considered more cost effective [17,18]. Therapeutic treatment for anthrax infection is based on antibiotic use, post exposure vaccination and anti-toxin antibodies, with a combinatorial approach of rapid post exposure vaccination combined with antibiotics treatment being the most promising [19]. Faced with the possibility of anthrax strains being engineered for resistance to current antibiotics, the need for alternative treatments grow.

Lactobacilli are Gram-positive bacteria constituting part of the normal oro-gastrointestinal flora [20] and generally regarded as safe (GRAS) for human consumption. Their ability to colonise and thrive in the gastrointestinal tract has directed attention to their potential use for therapeutic and prophylactic delivery of biomolecules [21]. Engineered lactobacilli have previously been used to deliver antibody fragments targeting both viral and bacterial infections [22,23]. ScFvs, while retaining the specificity of the monoclonal antibody from which they are derived, has a simpler structure, allowing production in bacterial expression systems. Several anthrax toxin neutralising scFvs have been derived from neutralising monoclonal antibodies or by panning of scFv libraries. The anti-anthrax PA scFv 1H is derived from the 14B7 monoclonal antibody through molecular evolution, yielding a highly stable scFv with increased binding affinity [24].

We have developed several recombinant lactobacilli expressing a single-chain antibody fragment (1H scFv) against the PA toxin, and tested their ability to provide passive immunity against anthrax toxin in the gastrointestinal tract.

## Results and discussion

### Construction of anti-PA expressing recombinant *Lactobacillus*

**Figure 1:**
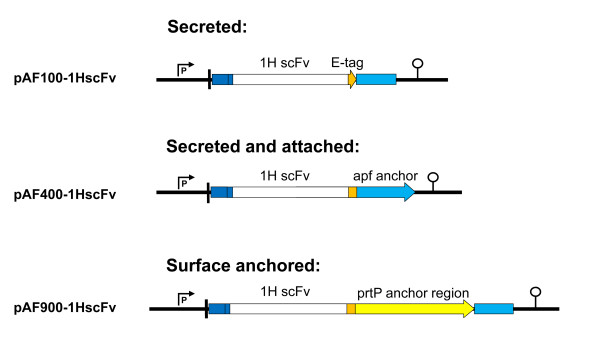
**Plasmid constructs for expression of the 1H scFv in lactobacilli based on the expression cassette from the *apf *gene from *Lactobacillus crispatus *M247**. Variations in the anchoring domain and placement of the translational stop codon gives respectively secreted, cell wall anchored or attached production of the scFv. The APF promoter (P), APF signal peptide (blue), APF anchoring domain (light blue), translational stop codon (arrowhead), 1H scFv (white), prtP anchor (yellow), E-tag (orange) and transcriptional terminator (lollipop) are indicated.

A series of anti-PA neutralising scFvs have previously been generated through random mutagenesis of the monoclonal antibody 14B7 [24]. The 1H scFv, had a K_d _of 0.25 nM, nine fold lower than the parent monoclonal antibody and provided protection both *in vitro *and *in vivo *[24]. To evaluate the therapeutic potential of *Lactobacillus *expressing a scFv against anthrax toxin, a series of expression cassettes was constructed with the 1H scFv encoding gene placed under the control of the *apf *promoter and fused to the *apf *signal peptide at the N-terminal and with a C-terminal E-tag for detection (Figure 1). Variations in the C-terminal parts of the plasmids gives rise to three different methods of production of the scFv. In pAF100-1HscFv, a stop codon just terminal of the E-tag leaves the scFv secreted into the media (referred to as a secreted construct), pAF900-1HscFv has the C-terminal E-tag fused to the prtP anchoring domain leading the scFv to be covalently bound and displayed on the cell wall upon secretion from the cell (referred to as an anchored construct), and lastly, pAF400-1HscFv, where the E-tag is fused to the anchoring domain of the *apf *gene which attaches the scFv non-covalently to the cell wall upon secretion, (referred to as an attached construct).

**Figure 2:**
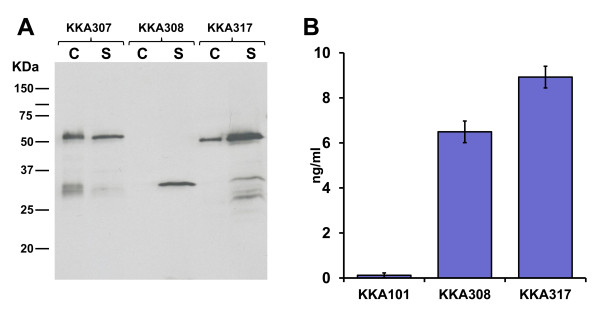
**(A) Detection of the scFv expressed by recombinant *L. paracasei *by immunoblotting**. Cell extract (c) of cell wall anchored strain (KKA307), secreted strain (KKA308) and attached strain (KKA317). Culture supernatant (s) from cell wall anchored strain (KKA307), secreted strain (KKA308) and attached strain (KKA317). The expected size of *L. paracasei *produced scFvs was 57.1, 29.2 and 42.2 KDa for the anchored, secreted and attached constructs respectively. (B) Binding and quantification of anti-PA scFv secreted into the growth media of the recombinant lactobacilli as measured by ELISA, with 1H scFv purified from *E. coli *as a reference (average of 4 experiments).

Expression and correct localisation of the three constructs upon transformation into *Lactobacillus paracasei *was verified by Western blot analysis of the supernatant and cell fractions of cultures grown in MRS (Figure 2A). The scFv expressed by both the secreted (KKA308) and anchored constructs (KKA307) were found primarily in the expected fraction, the supernatant for KKA308 and the cell fraction for KKA307. Some scFv were found in the supernatant fraction of the lactobacilli expressing the anchored construct, which is likely to be due either to saturation of anchoring sites or inefficient anchoring of the scFv. For lactobacilli expressing the attached construct, KKA317, the scFv was found to be bound to the cell wall but also secreted into the media in significant amounts. This is probably due to the weaker nature of the non-covalent binding to the cell wall of the APF binding domain. The total scFv production for the lactobacilli expressing the attached construct was 2-3 fold higher, relative to the two other constructs, despite being expressed from the same promoter. We have previously observed this effect for scFv fusions to the APF anchoring domain [25] suggesting that the fusion could be beneficial for the secretion or stability of the scFv fragments in the supernatant.

The three expression constructs provide a choice for the mechanism of neutralisation 1; anchored and attached scFv constructs immobilising PA on the cell wall of the lactobacilli and clearing of bound PA from the intestinal tract by gastric emptying 2; secreted scFv expression as seen both using the secreted construct and the attached construct where a significant proportion of the scFv are non-cell wall attached, leading to diffusion of the neutralising scFvs in the gastrointestinal lumen with subsequent binding and inactivation of PA.

### Binding activity of 1H scFv

Binding activity of the scFv from the culture supernatants from lactobacilli transformed with the secreted and attached constructs, was analysed by ELISA. ScFvs from both strains bound to PA coated microtiter plates (Figure 2B). Six and a half ng/ml (0.224 nM) and nine ng/ml (0.216 nM) was produced by the lactobacilli expressing the secreted and attached construct respectively, when quantified using a purified His tagged 1H scFv produced in *E. coli *as a positive control.

**Figure 3:**
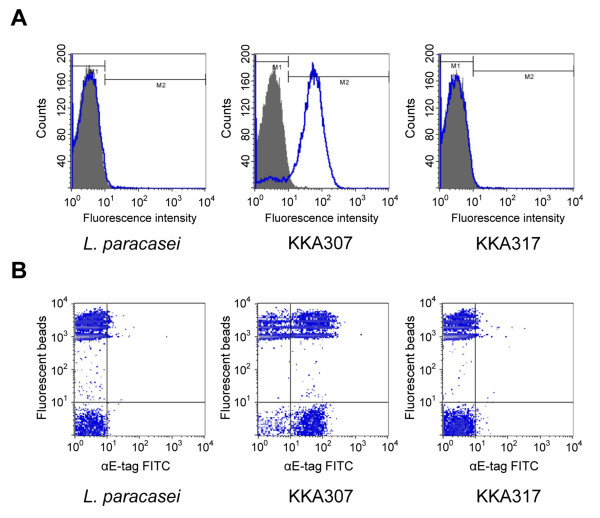
**Flow cytometry analysis of lactobacilli surface displayed scFvs**. (A) Visualisation of the production and display of the 1H scFv on the surface of recombinant *L. paracasei *presenting the scFv anchored (KKA307) or attached (KKA317) (wt *L. paracasei *was as a negative control). ScFvs were visualised by detection of the E-tag fused to the scFv using a mouse anti-E-tag antibody in conjunction with a FITC conjugated rabbit anti-mouse immunoglobulin antibody. (B) Binding of recombinant *L. paracasei *through surface displayed 1H scFv to PA coated fluorescent beads. The display of scFv on surface visualised through binding of mouse anti-E-tag antibody and FITC conjugated rabbit anti-mouse immunoglobulin antibody. 1,1 quarter: unlabeled *L. paracasei*, 2,1 quarter: scFv displaying *L. paracasei*, 1,2 quarter: PA conjugated fluorescent beads and 2,2 quarter: PA conjugated beads bound by *L. paracasei*.

Presence of the 1H scFv on the cell wall in the lactobacilli transformed with the anchored (KKA307) or attached (KKA317) construct was tested by flow cytometry (using staining with a mouse anti-E-tag antibody together with a FITC conjugated rabbit anti-mouse immunoglobulin antibody) (Figure 3A). For the anchored construct (KKA307), a strong positive signal confirmed the surface location of the scFv. Bacteria transformed with the attached construct (KKA317) did not stain, indicating that either the scFv is not displayed on the surface or that the E-tag is embedded in the membrane due to its close proximity to the anchoring domain and thus not accessible to the anti-E-tag antibody.

Using PA conjugated florescent beads, a strong binding to the KKA307 strain (displaying 1H scFv cell wall anchored), but not strain KKA317 displaying 1H scFv attached was observed (Figure 3B). The lack of binding observed with the lactobacilli expressing the attached construct might arise due to that the scFv are not protruding far enough from the cell wall to afford effective binding. We have recently shown that close proximity of the scFv attached with the APF anchoring domain may inhibit the binding activity of a cell wall anchored scFv [25]. Insertion of an spacer increasing the length of the anchoring domain could potentially resolve this as it has previously been shown by us to improve binding of cell wall display of antibody fragments [23].

### *In vitro *protection

**Figure 4:**
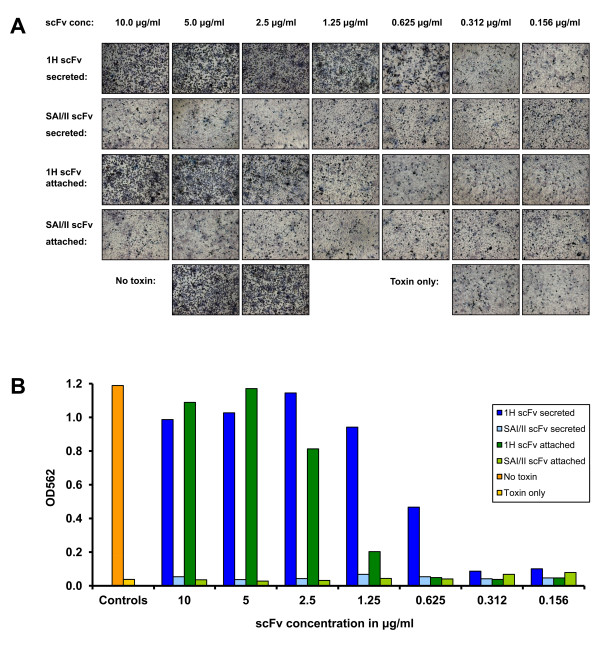
***In vitro *neutralisation of anthrax LT by purified *Lactobacillus *produced scFv**. (A) *In vitro *neutralisation by *L. paracasei *produced scFvs of anthrax LT in a macrophage toxicity assay (cell viability) visualised by staining with MTT. Comparison of neutralising capabilities of the secreted 1H scFv produced by KKA308 and the attached 1H scFv produced by KKA317 with the corresponding secreted and attached SAI/II as negative controls. (B) Quantification of *in vitro *neutralisation by colorimetric measurement of the break down of MTT into dark blue MTT-formazan.

The ability of the *Lactobacillus *produced anti-PA scFvs to protect against the toxin *in vitro *was assessed using the J774 MΦ cell line by exposing it to a lethal dose LT. Two fold serial dilutions of the 1H scFv, purified from the supernatant from the KKA308 (secreting) and KKA317 (attached) strains, were pre-mixed with the toxin complex (PA and LF) before challenge. A dose of 1.25 μg/ml of secreted scFv afforded complete protection, corresponding to a molar ratio of 3.5:1 of 1H scFv (29.2 KDa) to PA (83.3 KDa) (Figure 4B). The scFv from the lactobacilli transformed with the attached construct (42.2 KDa), afforded nearly full protection at a dose of 2.5 μg/ml and full protection was conferred at 5 μg/ml, translating to a molar ratio of 5:1 and 10:1 respectively. 1H scFv produced and purified from *E. coli *were also tested and showed protection at corresponding doses (data not shown) indicating that the binding affinity of the 1H scFv fragment is maintained when utilising the *Lactobacillus *based expression system.

### *In vivo *protection

To test the prophylactic effect of recombinant lactobacilli expressing the 1H scFv we developed a mouse model of oral challenge with *Bacillus anthracis *edema toxin (ET). ET was previously shown to cause massive fluid retention and swelling (edema) and intravenous injection shown to induce intestinal intralumenal fluid accumulation [13]. In our model of oral ET challenge a dose of 50 to 100 μg ET causes a significant fluid accumulation in the small and large intestine 16 hours post oral exposure leading to a 10-15% increase in total intestinal weight (Table 1). To mimic a stable colonisation achievable with good colonising bacterial strains the recombinant lactobacilli were given both 4 hours before and simultaneous with the toxin challenge. Mice receiving *Lactobacillus *expressing the attached construct (KKA317) and no ET were used as negative controls and had a median relative intestinal weight of 9.48% of the total body weight (Table 2). The groups receiving either toxin only or the non-protective *L. paracasei *pAF400 expressing attached a scFv against an irrelevant antigen (SAI/II from *S. mutans*) together with ET, had median relative intestinal weights of 10.67% and 10.99%, giving an increase in the median relative intestinal weight of 12.6% and 15.9% respectively (P< 0.05 for both). For the group treated with *Lactobacillus *expressing the 1H scFv in an attached form (KKA317), the median relative intestinal weight was 9.53% upon challenge with ET, i.e. in the same range as the negative control and significantly lower compared to mice receiving ET only (P< 0.05), indicating a blocking of the uptake of ET in the intestine. Mice treated with *Lactobacillus *expressing 1H scFv either in a secreted or anchored form together with ET, did not show any significant difference when compared to the ET only group (data not shown).

**Table 1:** Dose study of oral challenge with edema toxin

Oral dosage	n	Median	Minimum/maximum	**Relative median weight**^**a **^**(%)**	P value
No toxin	12	9.49	7.06/10.43	----	-----
10 μg ET	5	9.51	7.12/10.49	0.21	NS
25 μg ET	6	10.24	8.27/10.94	7.90	NS
50 μg ET	6	10.71	10.39/11.44	12.86	< 0.05^b^
100 μg ET	5	10.69	10.46/11.65	11.87	< 0.05^b^

Median (minimum and maximum) relative intestinal weight in percent total body weight upon challenge with increasing doses of edema toxin. ^a ^The difference in median of relative intestinal weight between experimental group and mice receiving no toxin in percent. ^b ^Represents a statistically significant difference of P< 0.05 compared with mice receiving no toxin using the Mann-Whitney U-test.

**Table 2:** Mouse model of oral challenge by edema toxin

Oral dosage	n	Median	Minimum/maximum	**Relative median weight**^**a **^**(%)**	P value compared to KKA317 only	P value compared to ET only
ET only	4	10.67	10.48/11.53	12.55	< 0.05^b^	----
KKA317 only (negative control)	5	9.48	7.13/10.51	----	-----	< 0.05^c^
KKA317 + ET	8	9.53	7.25/10.89	0.53	NS	< 0.05^c^
*L. paracasei *pAF400 + ET	3	10.99	9.99/11.05	15.93	< 0.05^b^	NS

Median (minimum and maximum) relative intestinal weight in percent total body weight upon challenge with either ET, recombinant *Lactobacillus *or both. ^a^ The difference in median of relative intestinal weight between group and negative control KKA317 in percent. ^b ^Represents a statistically significant difference of P< 0.05 compared with mice only receiving KKA317 using the Mann-Whitney U-test. ^c ^Represents a statistically significance of P< 0.05 compared with mice receiving only ET toxin using the Mann-Whitney U-test.

The reason why, in contrast to the attached construct, the secreted or anchored constructs failed to provide protection, remain to be elucidated. One explanation might be the dual function of the attached construct where the scFv is both cell wall displayed and secreted into the supernatant. The secreted part of the 1H scFv produced by the attached construct would have an unbound cell wall attaching domain, allowing it to re-attach to the cell wall of lactobacilli after binding to PA. This could theoretically provide a therapeutic advantage as the lactobacilli in the gastrointestinal flora could function as a binding reservoir for the attached 1H scFv, mopping up PA and immobilising it on the bacteria.

**Figure 5:**
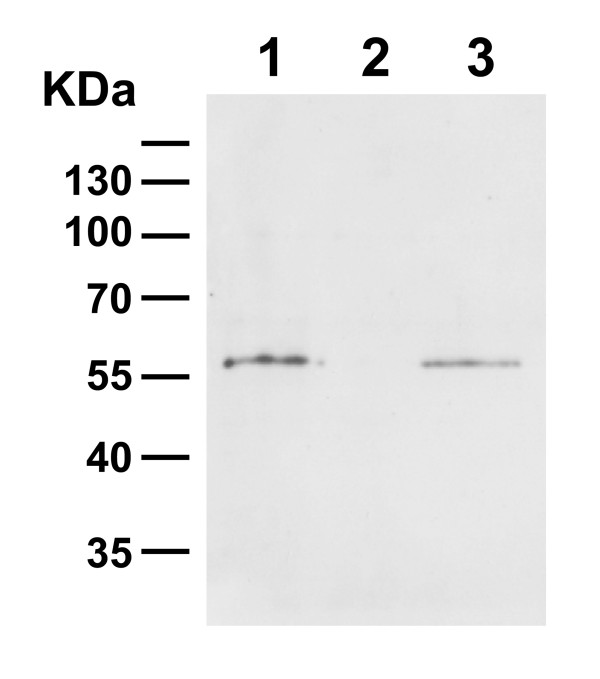
**Re-attachment of 1H scFv to the cell wall of *L. paracasei *detected by immunoblotting**. Pellet fraction of attached strain (KKA317) and (KKA101) grown in conditioned media containing 1H scFv (lane 1 and 3 respectively). Pellet fraction of strain KKA101 grown in conditioned media containing no 1H scFv, lane 2.

To examine if the attached 1H scFv could re-attach to the cell wall of lactobacilli we therefore grew a non-expressing strain of *L. paracasei *(KKA101) in media with and without the attached 1H scFv. Western blot analysis of the cell pellet of the cultures showed a clear re-attachment of the 1H scFv on the cell wall of the non-expressing strain (KKA101) when grown in media containing the scFv (Figure 5). Homologous binding domains are also found in other Gram-positive bacteria [26,27] but further studies would be needed to determine if re-attachment also occur on other bacteria and if this is a parameter for neutralisation with the attached construct.

However since only one of three constructs was successful in providing neutralisation *in vivo *certain issues still needs to be addressed for therapeutic engineering of lactobacilli for antibody expression. The length of the anchoring domain and polarity of the scFv might influence the extension of the scFv from the bacterial cell wall for and thereby affect binding. Stability of secreted scFvs might likewise be a determining factor for successful neutralisation and engineering of the scFvs for improved stability in the gastrointestinal lumen would likely improve the therapeutic effect [28,29].

The strategy of using lactobacilli for delivery of protective antibody fragments relies on their ability to thrive and colonise in the gastrointestinal tract. In the described study a engineered laboratory strain of *L. paracasei *were used as a proof of concept for mediation of *in situ *neutralisation in the gastrointestinal tract. For efficient continuous delivery of antibody fragments a *Lactobacillus *strain characterised for long-term colonisation in the host should be selected.

Recombinant lactobacilli have previously also been successfully used as a delivery system for oral vaccination with recombinant PA fused to a dendritic cell targeting peptide, giving a protective response four weeks after first oral dose [30]. Though effective, induction of a protective immune response might be too slow to provide protection in the case of an imminent risk of exposure. For effective protection a dual strategy of both passive immunity and oral vaccination could therefore be advantageous providing both rapid protection and protective immunity.

The use of toxin neutralising antibody fragments in the gastrointestinal tract can potentially be used as treatment against other pathogens like *Clostridium difficile, Vibrio cholera *and *E.coli O157:H*. Recently a neutralising single domain antibody fragment (VHH) against *Clostridium difficile *toxin A was developed [31] illustrating this approach. We have previously shown that VHH can be produced at high levels using the described expression system and in addition have advantages over scFvs for expression in lactobacilli [25], so as more VHH are being developed their use expressed from engineered lactobacilli for therapies in the gastrointestinal tract will likely increase.

## Conclusion

Our results demonstrate a possibility of employing a recombinant approach for neutralisation of bacterial toxins in the gastrointestinal tract as illustrated here, targeting anthrax toxins, using genetically engineered *Lactobacillus*. In the present study we have shown that a high affinity anti-PA scFv can be expressed both cell wall anchored and secreted by lactobacilli and retain its binding affinity. *In vivo *neutralisation was achieved in a mouse model of oral toxin challenge with engineered lactobacilli expressing the neutralising scFv with an APF anchoring domain. Using recombinant *Lactobacillus *for induction of passive immunity in the gastrointestinal tract as described in this study provides a possibility for a continuous delivery of the antibody *in situ *that can be used both therapeutically and prophylactically. The approach can also be extended to targeting of a range of toxins produced by a variety of gastrointestinal pathogens.

## Materials and methods

### Bacterial strains, plasmids and growth conditions

*E. coli *DH5α (Invitrogen, Carlsbad, CA) was grown in LB media at 37°C with 220 rpm orbital shaking or on LB-agar plates at 37°C. Lactobacilli were grown in lactobacilli MRS broth (Difco, Sparks, MD) at 37°C without agitation or anaerobically on MRS-agar plates (BD - GazPak EZ, Sparks, MD). Antibiotics were added at the following concentrations when indicated: ampicilin (100 μg/ml) and erythromycin (300 μg/ml *E. coli *and 5 μg/ml lactobacilli).

### Construction of recombinant *Lactobacillus* and *E. coli* strains

The 1H scFv was amplified from the pMoPac16 vector containing the 1H scFv [24] using the primers; anthrx1H-Fw: 5''-CCGGCCATGGATGATATTCAGATGACACAGACTAC-3'' and anthrx1H-Rv: 5''-GCACCTGCGGCCGCCGAGGAGACGGTGACTGAG-3''. The PCR fragment was cloned into pGEM^^®^^-T easy vector (Promega, Madison, WI) and DNA sequence verified by sequencing. The 1H scFv gene was excised using *Nco*I and *Not*I restriction enzymes (Promega) and ligated into the *Nco*I/*Not*I digested *Lactobacillus *expression vectors, pAF100, pAF400 and pAF900 [25] giving plasmids pAF100-1HscFv, pAF400-1HscFv and pAF900-1HscFv for secreted, attached and anchored expression respectively. The expression plasmids were transformed into *L. paracasei *(previously known as *L. casei *or *L. zeae *ATCC 393 pLZ15^- ^[32]) by electroporation as previously described [23,33], generating the *Lactobacillus *strains KKA307, KKA308 and KKA317 expressing the 1H scFv anchored, secreted and attached respectively. A *Lactobacillus *strain, KKA101, harboring a non-expressing version of the plasmid was constructed by transforming *L. paracasei *with the empty pIAV7 plasmid [34]. The *Lactobacillus *strain *L. paracasei *pAF400, expressing an attached scFv against an irrelevant antigen (SAI/II from *S. mutans*) has been described previously [25].

An *E. coli *strain for periplasmic expression of the 1H scFv was constructed by amplifying the scFv fused to the E-tag from pAF900-1HscFv with primers anthrx1H-pOPE-Fw: CGGCCATGGCGGATATTCAGATGACACAGACTAC and pOPE-Etag-Rv: CCGTATCCGGACCCGCTGGAACCGCGTCATCATCACCATCATCAT**TAA**TCTAGAGCC. The PCR fragment was restriction digested with *Nco*I and *Bgl*II (Promega) and cloned into the *Nco*I/*Bgl*II digested plasmid pOPE101-215(yol) [35] generating pOPE101-1HscFv(E-tag). The plasmid was transformed into *E. coli *XL1-Blue competent cells (Agilent, Santa Clara, CA) by electroporation generating the strain KKA300 and the DNA sequence verified by sequencing.

### Western Blot

The transformants were grown in MRS with 5 μg/ml erythromycin until an OD600 of 1.0. The cultures were centrifuged at 3,200 × *g *to separate the pellet from the supernatant. The supernatant was filter sterilised, pH adjusted to 7.0, dialysed against 10 mM Tris (pH 8.0) and concentrated using Amicon Ultra-4 centrifugal filter units (10 kDa cut off, Millipore, Carrigtwohill, Co. Cork, Ireland). The concentrated supernatant was mixed with 2 × Laemmli buffer and boiled for 5 minutes (min). The cell culture pellet was washed twice with PBS, resuspended in 100 μl Laemmli buffer and boiled for 5 min. The cell extract was centrifuged at 16,000 × *g *to remove cell debris and the supernatant containing soluble proteins was kept. The supernatant and cell extract were run on a 10% SDS-polyacrylamide gel at 170 volts and the proteins were transferred onto a nitrocellulose membrane (Hybond-ECL, GE Healthcare, Little Chalfont, Buckinghamshire, UK). The membrane was blocked with PBS-T (PBS with 0.05% (v/v) Tween 20 + 5% (w/v) milk powder) and successively incubated with mouse anti-E-tag antibodies (1 μg/ml, GE-Healthcare) and HRP (horse radish peroxidase) labelled goat anti-mouse antibodies (DAKO A/S, Glostrup Denmark). The signal was detected by chemiluminescence using the ECL Plus™ Western Blotting detection system (GE Healthcare).

For re-attachment of 1H scFv on lactobacilli, strains KKA317 and KKA101 were grown in 50 ml MRS with 5 μg/ml erythromycin until OD600 of 1.0. Cultures were harvested by centrifugation and supernatant filter sterilised and adjusted to pH 7.2. The conditioned media were re-inoculated with KKA317 and KKA101 at an OD600 of 0.2 and grown to OD600 of 1.0. Cell pellets were treated as previously described and run on an 10% SDS-polyacrylamide gel and Western blotted.

### Enzyme-Linked ImmunoSorbent Assay (ELISA)

96 well microtiter plates (EIR/RIA plate, Costar, Lowell, MA) were coated with 100 μl rPA (List labs, Campbell, CA) at 1 μg/ml in PBS overnight (o/n) at 4°C. Plates were subsequently blocked with 200 μl 1% BSA (in PBS containing 0.05% Tween 20, PBS-T) for two hours at 4°C. After washing with PBS-T, dilutions of scFv producing *Lactobacillus *culture supernatants were added and the plates incubated at 4°C o/n. ScFvs purified from *E. coli *were used as a positive control for quantification. Plates were subsequently washed three times and 100 μl mouse anti-E-tag antibody (GE-healthcare) was added (1 μg/ml) in blocking solution, followed by incubation at room temperature for 2 h. Plates were then washed three times in PBS-T and incubated with 100 μl AP conjugated rabbit anti-mouse antibody at 1/1000 (Dako A/S, Glostrup Denmark) in blocking solution. Following an additional 1 hour incubation at room temperature, the plates were washed twice in PBS-T and once in PBS, resuspended in 100 μl of diethanolamine buffer (1M, pH 10.0) containing 1 mg/ml pNPP (Sigma-Aldrich, St. Louis, MO) and absorbance was read after 10-30 min at 405 nm in a Varioskan Flash (Thermo Scientific, Waltham, MA).

### Flow cytometry

50 μl of *Lactobacillus *cultures grown to an OD600 of 1.0 in MRS were harvested by centrifugation (8000 rpm, 1 min) and washed three times in PBS. Bacteria were resuspended in 50 μl PBS with 1% BSA (PBS-BSA) and incubated for 30 min on ice sequentially with 30 μl PA coated beads (Invitrogen), 50 μl anti-E-tag antibody (10 μg/ml) and 50 μl FITC conjugated anti-mouse immunoglobulins (diluted 1/100) (Jackson Immunoresearch Laboratories, West Growe, PA), all diluted in PBS-BSA. Bacteria were washed with 500 μl PBS between all three incubations. Samples were resuspended and fixed in 300 μl 2% paraformaldehyde in PBS and analysed using a FACS Calibur machine (Becton Dickinson, Franklin Lakes, NJ). 1 μm red FluoSpheres^^®^^microspheres (Invitrogen) were incubated with rPA (List labs) according to manufactors instructions to generate PA coated fluorescent beads for use in flow cytometry.

### Macrophage toxicity assay to assess neutralisation by scFvs

Protection by *Lactobacillus *and *E. coli *produced scFvs were analysed by their capacity to protect the J774 MΦ cell line from killing by LT [36,37]. Briefly, J774 MΦ were added to 96-well, flat-bottom wells (5 × 10^4 ^MΦ/well) and incubated at 37°C in 5% CO_2 _in air. After 12 hours of incubation, LT (i.e., 1 μg/ml rPA and 1 μg/ml LF, (List labs)) pre-mixed with scFvs were added to the cultures and incubated for an additional 12 hours. Viable MΦ were evaluated by colorimetric assay by reading absorption at 562nm after addition of Methylthiazolyldiphenyl-tetrazolium bromide (MTT) (Sigma-Aldrich) [38]. MTT was used at a concentration of 5 mg/ml, and a volume of 20 μl (100 μg/well) was added to individual wells.

### Purification of scFvs

ScFv was purified from the supernatant of strains KKA308 and KKA317, grown in defined minimal media [39]. The scFv was isolated on a HiTrap™ anti-E-Tag Column (GE-healthcare) according to the manufactures instructions. Eluate was concentrated on Amicon Ultra-4 10K MWCO spin column (Millipore, Billerica, MA). The concentration of purified scFv was determined using the Micro BCA™ Protein Assay kit (Pierce, Rockford, IL) with BSA as a standard.

The 1H scFv was isolated from the periplasma of the recombinant *E. coli *strain, KKA300, as previously described [35] with the following modifications. The culture was grown in 500 ml YT-broth supplemented with 100 mM glucose and 100 μg/ml ampicilin. The periplasmic extract dialyzed against PBS was adjusted to 30 mM imidazole (Sigma Aldrich) and 0.5 M NaCl (pH 7.5). The adjusted periplasmic extract was immobilised on a 5 ml HisTrap™ HP Column (GE-healthcare) and washed with 20 bed volumes wash buffer (PBS, 30 mM imidazole, 0.5 M NaCl, pH 7.5) and subsequently eluted with 5 bed volumes elution buffer (PBS, 0.5 M imidazole, 0.5 M NaCl, pH 7.5). Eluate was concentrated and buffer exchanged with PBS on a Amicon Ultra-4 10K MWCO spin column (Millipore, Billerica, MA) and purified scFv concentration determined as described above.

### *In vivo* neutralisation

Female C57BL/6 mice, six-seven weeks of age, were obtained from Jackson Laboratories (Bar Harbor, ME). Mice were maintained under specific pathogen-free conditions and provided food and water *ad libitum*. All studies were performed in accordance with both National Institutes of Health and Institutional guidelines and approved by the Ohio State University Institutional Animal Care and Use Committee (Protocol number 2009A0210).

A dose study of the oral effect of ET was carried out on groups of mice challenged with 10, 25, 50 and 100 μg of ET (equal amount of rPA plus EF (List Labs)) given in 100 μl PBS by gavage. After 16 hours, the toxic effect was measured as ET induced fluid accumulation in the small and large intestine. Mice were euthanized with CO_2 _and death confirmed by cervical dislocation prior to removal of small and large intestine. Fluid accumulation was measured as percent of the weight of the small and large intestine compared to total body weight.

The KKA307, KKA308, KKA317 and *L. paracasei *pAF400 [25] strains were grown in MRS to an OD600 of 1.0, harvested by centrifugation and resuspended in culture supernatant with pH adjusted to 7.0 to give 5 × 10^9 ^cfu/ml. Nine-twelve weeks old C57BL/6 mice (body weight 15-20 g) were given 2.5 × 10^9 ^cfu recombinant *Lactobacillus *by gavage. Four hours later they were challenged with a non-lethal dose of 50 μg ET (50 μg rPA plus 50 μg EF (List Labs)) together with an additional 2.5 × 10^9 ^cfu recombinant *Lactobacillus *by gavage. After 16 hours, the toxic effect of the ET was measured as fluid accumulation in the small and large intestine.

### Statistical analysis

The relative intestinal weight, in percent of total body weight of the treated groups, were compared to mice group receiving ET only, and analysed with the Mann-Whitney U-test using the GraphPad Prism software.

## Competing interests

The authors declare that they have no competing interests.

## Authors' contributions

KKA constructed recombinant *Lactobacillus *and *E. coli*, purified scFvs, carried out expression analysis and *in vitro *protection assays, designed experiments and drafted the paper. HM and LH developed the experimental strategy. BA assisted protein purification. PB carried out the *in vitro *protection assays and *in vivo *model.

All authors read, revised and approved the manuscript.
